# Multiple group membership and executive function in a socioeconomically diverse sample

**DOI:** 10.1038/s41598-024-60534-4

**Published:** 2024-04-30

**Authors:** Nobuhiko Goto, Sony Kusumasondjaja, Fandy Tjiptono, Shirley X. L. Lim, Dexter Shee, Aya Hatano, Nuri Herachwati, Alexandre Schaefer

**Affiliations:** 1 Graduate School of Social Sciences, Hitotsubashi University, Tokyo, Japan; 2 Department of Management, Faculty of Economics and Business, Airlangga University, Surabaya, Indonesia; 3 School of Marketing and International Business, Victoria University of Wellington, Wellington, New Zealand; 4 Centre Des Sciences du Goût et de L’Alimentation, CNRS, INRAE, Université de Bourgogne, F-21000 Dijon, France; 5 Department of Psychology, Jeffrey Cheah School of Medicine and Health Sciences, Monash University Malaysia, Subang Jaya, Malaysia; 6 IdeaLab Inc., Tokyo, Japan; 7 Department of Psychology, School of Medical and Life Sciences, Sunway University, Petaling Jaya, Malaysia; 8 Present Address: Division of Psychology, Department of Clinical Neuroscience, Karolinska Institutet, Stockholm, Sweden

**Keywords:** Human behaviour, Cognitive control

## Abstract

Belonging to multiple groups is an important feature of our social lives. However, it is largely unknown if it is related to individual differences in cognitive performance. Given that changing self-identities linked to each group requires cognitive operations on knowledge bases associated with each group, the extent to which people belong to multiple groups may be related to individual differences in cognitive performance. Therefore, the main objective of this study was to test if multiple group membership is related to executive function task performance. A socioeconomically diverse sample of 395 individuals in Indonesia participated in this study. Our results show that multiple group membership was positively related to the 3-back working memory performance. However, we also found that this relationship was significant only among participants with high (not median or low) SES. We also observed that Contact diversity was negatively related to working memory performance among participants with low SES. Our results show that the complexity of our social lives is related to individual differences in executive function performance, although this seems to be constrained by SES.

## Introduction

Belonging to a variety of social groups is an important feature of our social lives. An emerging field of research has investigated the construct of multiple group membership (MGM), the extent to which individuals belong to multiple groups^1,2^. Past studies on MGM reported positive relationships between this construct and self-esteem^3^, resilience against physical pain^4^ and against negative life events^5^, well-being^6^, and retirees’ subjective health across cultures^7^ (see reviews^1,8^).

Despite the importance of MGM in these psychological and health domains^9^, very little is known about how cognitive function is related to belonging to multiple groups itself. Many of its features, however, suggest that it may be linked to individual differences in a key cognitive process—executive function (EF). More specifically, according to social identity theory^10^ and self-categorization theory^11^, parts of our self-concepts are constructed by social identities linked to every group to which we belong and to specific knowledge bases (e.g., semantic knowledge, autobiographical memories, goals and behavioural scripts). Therefore, belonging to multiple groups may involve frequently "updating" these knowledge bases as different social identities are made salient. Social identity salience refers to the extent to which people see themselves as a member of a given group in the context at hand^12^. When a certain social identity is made salient, people are cognitively and affectively tuned to that group’s norms and come to think and behave like members of that group. For example, think of an Asian American girl who is raised in an Asian family and attends a school where most students are Westerners. At home, she behaves as an Asian child and therefore she guides her behaviour from several cultural norms that include refraining from challenging authority figures and suppressing the expression of individual opinions that may be contrary to the group’s opinion^13^. At school, amongst Western children and teachers, she inhibits these social norms and adopts more Western social rules, which leads her to assertively speak up her opinions, even the most dissenting ones. More generally, when individuals are within the context of a specific social group, it may be necessary for them to inhibit irrelevant representations and goals related to other social groups, while they selectively activate representations and goals relevant to the current group. As soon as they become (implicitly or explicitly) aware of being in another group setting, they again have to dynamically update which representations are suppressed and activated^14^.

Such cognitive processes have a noticeable overlap with executive function processes, a well-known category of higher-order cognitive processes responsible for coordinating other cognitive processes in order to achieve specific goals^15–17^. A specific EF subprocess, the process of *working memory updating,* seems to overlap with the cognitive requirements of having MGM. This process involves both selecting and maintaining task-relevant contents in working memory (WM), and dynamically suppressing and replacing them with other contents when task demands have changed^18^. Past research has found a positive association between measures of MGM and performance in tasks assessing creativity^19^, and the six-item screener, a small questionnaire that includes a word recall task^20^. Although these studies indicate that MGM is related to general cognitive outcomes, it remains untested whether MGM is specifically related to EF task performance.

The main goal of the present study was to address this gap by testing whether MGM correlates with individual differences in EF. We examined this question while controlling for the diversity of social contacts. This construct is also termed as cross-group friendships or intergroup contacts^21^, and defined by the frequency of interactions that an individual has with people from a different group, and in particular people with different ethnic backgrounds. The number of social contacts in general has been shown to be linked to better memory and cognitive function (e.g.^22^), although it is largely unknown if it is related to EF. Belonging to different groups may be mutually related to more contact with people from different ethnic backgrounds; however, these are not fully overlapping factors, as MGM can refer to groups that are not organized alongside ethnic divisions, and interactions with someone from a different group does not necessarily imply belonging to this group, in which an intergroup contact is a case. Past studies on MGM have shown that general contacts (i.e., friends and relatives) did not predict cognitive functioning after controlling for MGM^20,23^. In line with these studies, we expected that Contact diversity would not be related to EF performance when MGM was controlled for.

We also exploratorily examined a moderating role of SES. More specifically, we used a moderation analysis to see if the correlation between MGM and EF is specific to a particular SES group or general across both groups. The primary goal of this analysis was to provide a methodological control for individual differences in SES, a factor that is crucial to EF performance as there is growing evidence that high SES people and low SES people are distinct groups in terms of their EF processes and performance^24,25^. This is likely to be even more relevant in diverse, non-WEIRD samples such as the one used here. Therefore, we wanted to ascertain that potential correlations involving EF were not biased by a lack of considering distinct groups of SES. Given that the goal of this analysis was primarily to provide a methodological control, we did not have strong a priori hypotheses on how SES could moderate the MGM-EF relationship, but several studies suggest that factors specific to low SES people may weaken the correlation between MGM and EF for low SES people, such as the amount of stress they experience^26,27^, lack of cognitive stimulation apart from belonging to groups^28^, and incompatibility of newly joined groups with their social background^29^. For this analysis, we chose to test a moderation model because of previous evidence suggesting that high and low-SES individuals may form distinct groups regarding cognitive and behavioural tasks (e.g.^25,30^). For completeness, we also tested the moderation analysis of Contact diversity-EF relationship. As mentioned above, we expected that Contact diversity would not be related to EF performance, and this was another methodological check to make sure that a potential contact-EF correlation would not be masked by omitting to consider differences between SES groups.

## Overview of the study

In summary, the primary goal of this study was to test if multiple group membership is related to EF performance. The secondary goal was to examine whether this relationship was not confounded by Contact diversity and whether the associations of MGM and Contact diversity were moderated by SES. Finally, we also took the opportunity to test our hypothesis in an Asian sample with a large variation in SES in order to operationalize SES in optimal conditions and adequately examine a moderating role of SES. Past research of MGM on cognitive functioning has been done exclusively with Western samples from economically developed countries^19,20^, and therefore testing a non-Western sample with large levels of socioeconomic variability allows to expand the diversity of samples tapped in this research domain, a task that is increasingly necessary in psychological research^31^.

To tackle these goals, we accessed a community sample with a wide variability in SES in the city of Surabaya, a large urban center in Eastern Java, Indonesia. MGM, SES and Contact diversity were assessed through individual structured interviews, and EF was measured through an N-back working memory task, a canonical EF task tapping WM updating processes^16,32^. Owing to the cognitive processes implied by the construct of MGM, we hypothesized that MGM would be related to an increase in EF performance over and above Contact diversity. We also explored the moderating role of SES in the relationship of MGM and Contact diversity with EF performance.

## Results

### N-back performance

**Figure 1:**
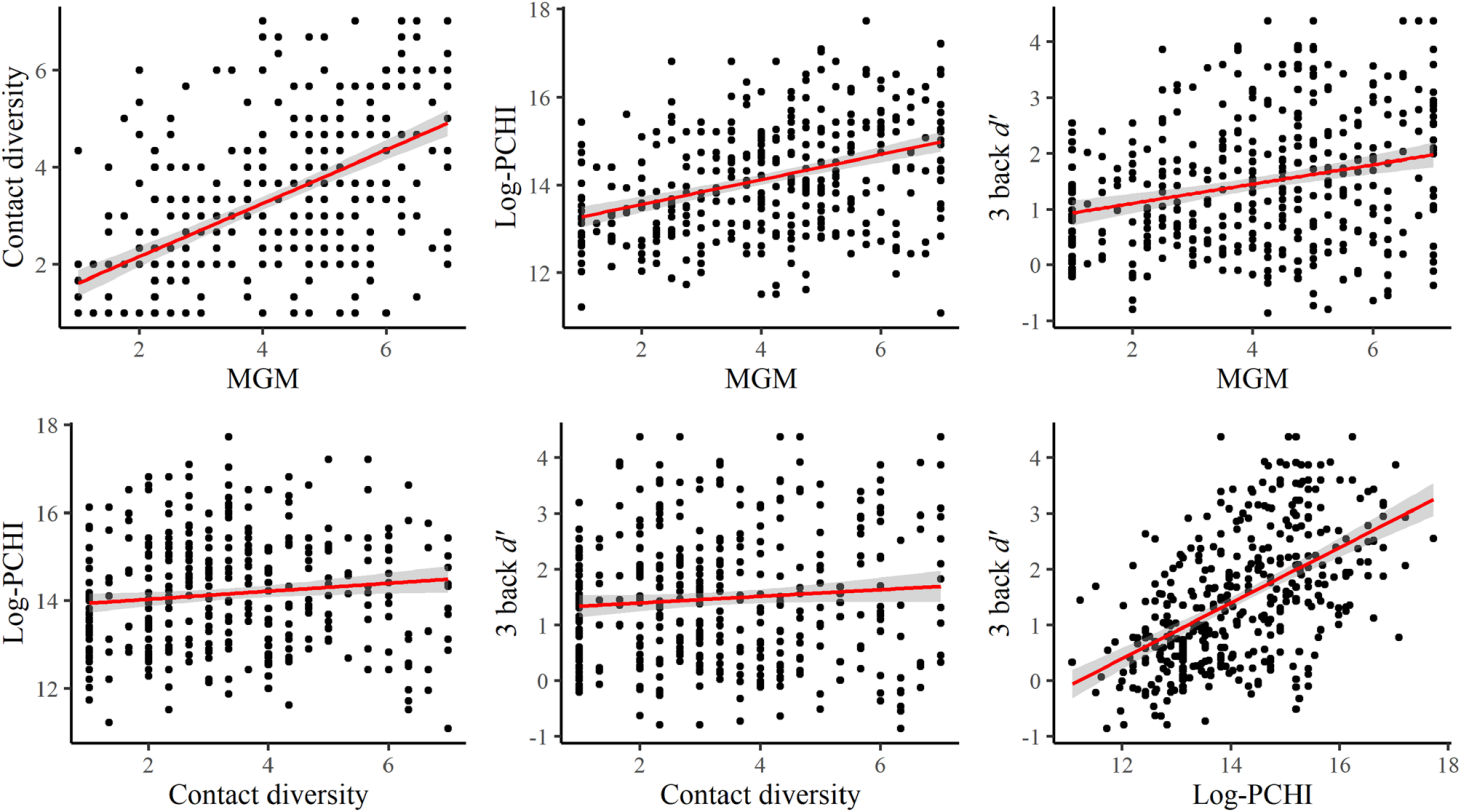
Scatterplots of key variables.

In order to ensure that analyzed data came only from participants who understood the task requirements and actively engaged with it, we excluded both those who had less than 36 (a half of all the trials) valid trials in the 3-back task or those whose accuracy performance suggested that the task requirements were not understood (general accuracy below 30%), as explained in the Participants section. The mean *d’* of 1-back (*n* = 394) was 2.44 (*SD* = 1.14) and of 3-back (*n* = 395) was 1.47 (*SD* = 1.18), *t* = 16.54, *p* < 0.001. The mean response time (RT) for correct trials of 1-back was 780 ms (*SD* = 208) and of 3-back was 861 ms (*SD* = 209), *t* = − 8.38, *p* < 0.001. Correlations between each variable and means and standard deviations are reported in Table 1 and scatterplots of four key variables are depicted in Fig. 1.

**Table 1:** Correlations, means, and standard deviations.

	3 back *d’*	MGM	Contact diversity	Log-PCHI	College enrollment	Age	Gender	3 back RT
MGM	0.26***							
Contact diversity	0.09	0.58***						
Log-PCHI	0.54***	0.39***	0.12*					
College enrollment	0.53***	0.43***	0.17***	0.76***				
Age	− 0.25***	− 0.15**	− 0.12*	− 0.16**	− 0.26***			
Gender	0.21***	0.04	0.01	0.22***	0.25***	− 0.08		
3 back RT	0.04	− 0.05	− 0.16**	0.01	− 0.08	0.27***	− 0.03	
Mean (*SD*)	1.47 (1.18)	4.08 (1.77)	3.31 (1.68)	14.15 (1.28)	0.54 (0.50)	35.14 (13.06)	0.42 (0.49)	0.86 (0.21)

p* < .05, p** < .01, p*** < .001.

Consistent with our hypotheses, 3-back *d’* was positively correlated with MGM (*r* = 0.261, *p* < 0.001), but not with Contact diversity (*r* = 0.085, *p* = 0.091). Log-transformed PCHI, college enrollment, age and gender were also correlated with 3-back *d’* (see Table 1). While visual inspections of Q-Q plots suggested that MGM, Contact Diversity, and mean SES may have violated the assumption of normality, Spearman’s “Rho” correlations indicated that the correlations remained similar to those obtained with Pearson’s. The Q-Q plots and Spearman’s correlation results can be obtained within the analysis script at OSF (see the Method section for the link). Next, to test whether MGM had a unique relationship with 3-back performance controlling for other variables, we first computed a multiple regression with 3-back performance as the dependent variable, and MGM, Contact diversity, age and gender as predictors in the same equation (Table 2). Visual inspections of Q-Q plots for this model, as well as for the other models reported below, confirmed that residuals were normally distributed (see the analysis script at OSF). The overall model was significant, *R*^*2*^*adj* = 0.144; *F*(4, 390) = 27.5, *p* < 0.001, VIF < 1.52. The results indicated that MGM was positively related to 3-back performance while Contact diversity showed a marginal negative relation.

**Table 2:** Multiple regression analyses with covariates.

	b	se	Beta	t	95%CI	*p*
(Intercept)	1.403	0.230		6.099	0.951, 1.855	< .001
MGM	0.188	0.038	0.282	4.915	0.113, 0.263	< .001
Contact diversity	− 0.072	0.040	− 0.103	− 1.807	− 0.151, 0.006	.072
Gender	0.428	0.111	0.180	3.844	0.209, 0.646	< .001
Age	− 0.018	0.004	− 0.202	− 4.267	− 0.027, − 0.010	< .001

**Figure 2:**
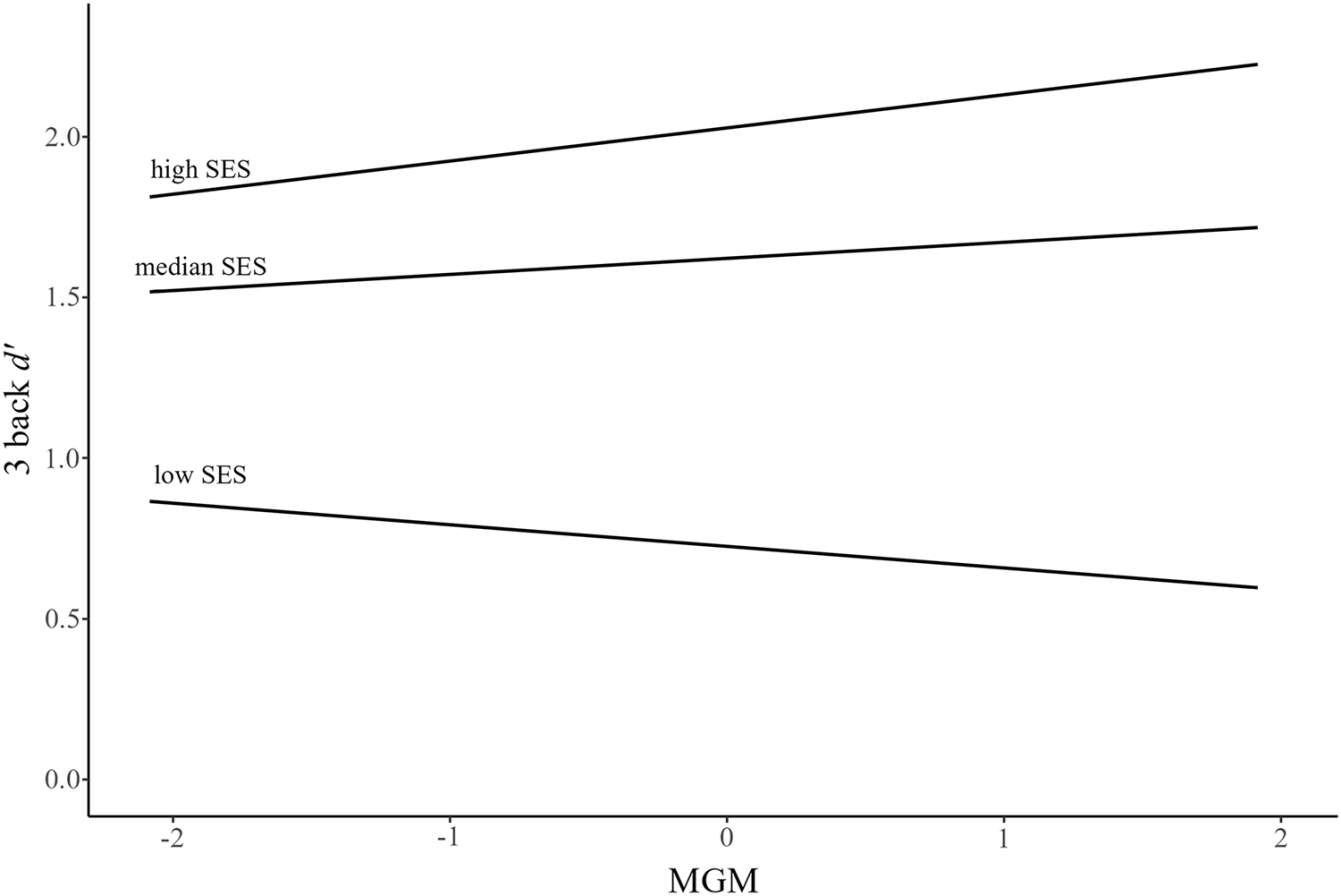
Multiple group membership and 3 back *d’* scores as a function of SES.

Next, we explored the moderating effects of SES separately using the PROCESS macro for R ^33^. In this analysis, we tested two different combinations: interactions between MGM and SES and between Contact diversity and SES. When the interaction with MGM was examined, Contact diversity was entered as a covariate and vice versa. Age and gender were entered in all analyses as covariates. Moderating effects of SES were examined at three levels: “median SES” indicates that the value of mean SES is at the 50th percentile, “high” SES means 84th percentile, and “low” SES means 16th percentile^33^. First, there was a significant moderating effect of SES with MGM (*b* = 0.085, *p* = 0.007), indicating that MGM was positively related to 3-back performance among high SES (*b* = 0.103, *p* = 0.034) but not median or low SES participants (*b* = 0.050, *p* = 0.197; *b* = − 0.067, *p* = 0.162, VIF < 1.86, Fig. 2). The analysis of Johnson-Neyman significance region suggested that the association was significant for participants who are in the top 28.86% of SES.

**Figure 3:**
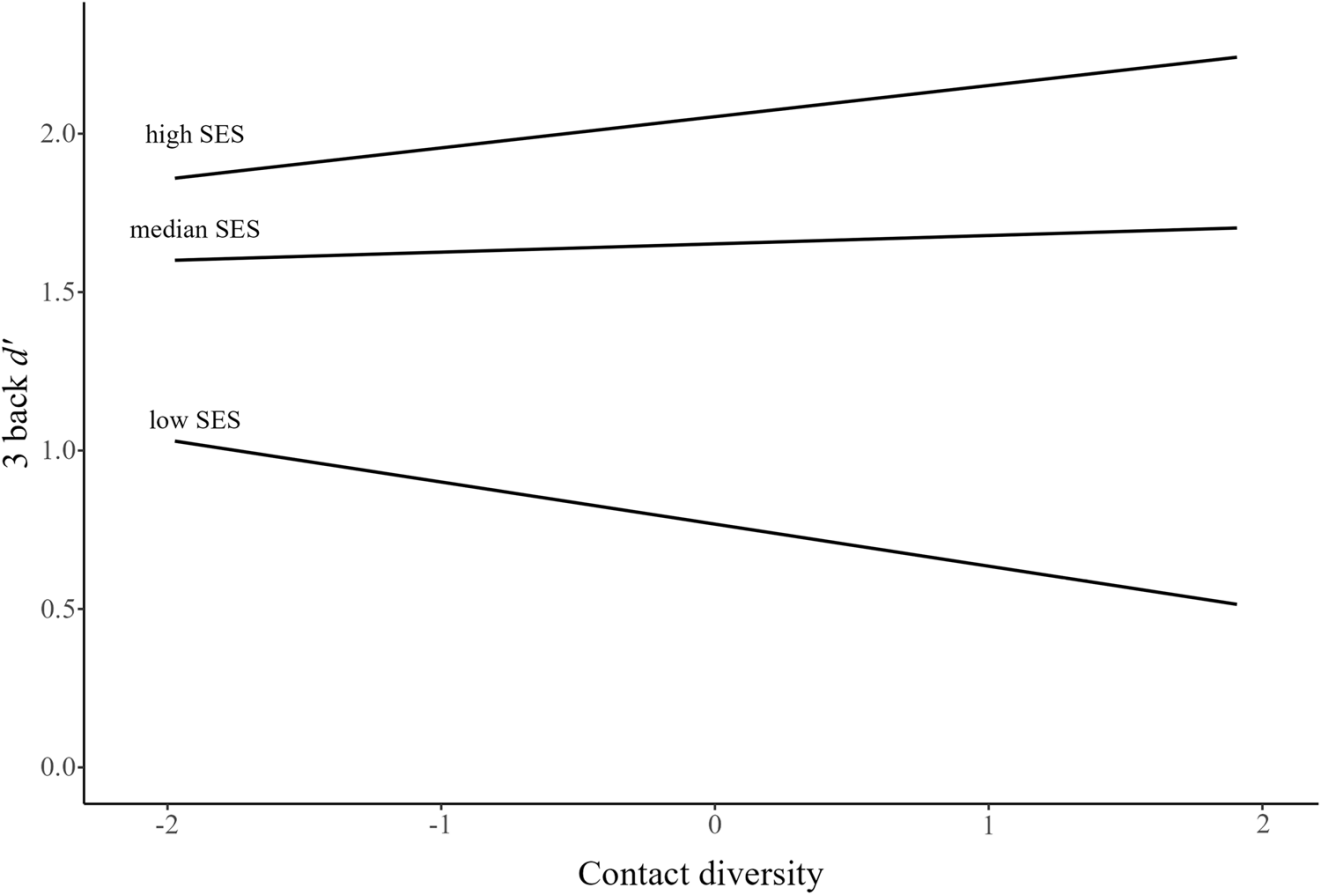
Contact diversity and 3 back *d’* scores as a function of SES.

Second, there was also a significant moderating effect of SES with Contact diversity (*b* = 0.115, *p* < 0.001), indicating that Contact diversity was negatively related to 3-back performance among low SES (*b* = -0.133, *p* = 0.004) while positively related among high (*b* = 0.098, *p* = 0.036), and not significantly related among median (*b* = 0.026, *p* = 0.711, VIF < 1.89, Fig. 3) SES participants. The analysis of Johnson-Neyman significance regions suggested that the association was significant for participants in the bottom 42.28% and top 21.27% of SES.

Consistent with our predictions, these results demonstrate that MGM was positively related to performance on a canonical EF task, the 3-back working memory task, above and beyond age, gender, and Contact diversity. However, such association was conditional on the levels of SES. Specifically, MGM was positively related to EF performance only among high SES. Furthermore, the present study also unexpectedly found that Contact diversity was negatively related to EF performance among low SES. The current results also replicated previous results about the positive relationship between SES indicators and EF^24^ and also found that SES was positively related to MGM and Contact diversity.

## Discussion

The main finding of this study is that individuals who belong to multiple social groups tend to have a strong performance in a task measuring a core cognitive capacity—executive function—while controlling for other related variables. More specifically, we found that MGM and two indicators of SES (income and education), but not Contact diversity, were significantly correlated with behavioural performance on a canonical EF task, the 3-back working memory task. A multiple regression showed that MGM had a unique positive association with EF, above and beyond Contact diversity, age, and gender. Furthermore, unlike past studies which examined the relationships between MGM and cognitive performances, the present study for the first time revealed that SES plays moderating roles between MGM and EF and between Contact diversity and EF. That is, MGM was positively related to EF among high SES individuals (those who attended a college and with high income per capita) but not related among low or median SES individuals (those who did *not* attend a college and/or with middle or low income per capita). On the other hand, Contact diversity was negatively related to EF among low SES while positively related among high SES individuals. These results provide a new picture of how MGM, Contact diversity, and SES are related to EF. In addition, we found that SES correlated with both MGM and Contact diversity. We discuss hereafter the potential explanations for these findings.

First, predictions derived from social identity and self-categorization theories posit that when a certain social identity is made salient by the context at hand, people are more likely to express thought, feelings, and behaviours that are prototypical of that identity^34^. This implies that belonging to multiple groups requires constant “updating” of different identities related to each group, and therefore the updating of knowledge bases related to these identities. Therefore, a high level of MGM is likely to involve EF processes needed to dynamically "update" self-representations and behavioural schemata that are relevant to a specific social group for a particular social context. Research on social identity and intergroup relations has been well aware of two social phenomena. First, people belong to multiple groups and, second, their cognitive responses would be very different depending on their salient social identities, which further leads to different emotions and behaviours^14^. The current study linked these two well-known observations in the literature and demonstrated for the first time that people who belong to more groups tend to also be individuals who have a better EF task performance. These results expand the social identity research by suggesting that social identity changes is related to EF, and in particular, its updating component.

Second, why was the MGM-EF relationship significant only for high-SES individuals? Here, it is important to note that the primary goal of these moderation analyses was to provide a methodological check of whether correlations with EF were not biased by the omission of considering distinct groups of SES. Therefore, we did not have strong hypotheses and the following interpretations are tentative, and provided solely for the purpose of generating ideas for future research. Taking this into account, updating and relevant changes in cognition, emotions, and behaviours discussed above may occur only if individuals strongly identify with their groups, internalize their identities, and value their memberships^35^. High SES individuals are more likely to have larger resources to choose their own memberships and identities^29,36,37^, which in turn results in valuing and expressing their memberships more. Low SES people may show equal or stronger group identification with their group especially when a group they belong to is a minority or a pervasively discriminated group^38^. This may be, however, limited to their “core” group (e.g., ethnicity). As low SES people increase the number of groups they belong to, their group identification with newly added groups may become weaker compared to high SES people. This is because, first, being a member of multiple groups is costly^37^, which would financially distress low SES people especially. Such distress is directly related to lower EF performance^26,27^ and may also lead to lower group identification. Second, when belonging to a new group involves upward mobility (e.g., being a first university student in the family or gaining high-paying employment), low SES people only weakly identify with the group because the group is incompatible with their social background^29^. It could be speculated that when group identification is low, even a high level of MGM would not involve EF processes to update behavioural schemata and goals relevant to specific social groups. These differences may have resulted in differences in relationships between MGM and EF among low and high SES individuals. Since this study did not measure group identification and the moderation analysis was rather exploratory, these interpretations are speculative and future research is needed to better elucidate these phenomena.

Third, why was Contact diversity negatively related to EF among low SES individuals? As mentioned above, it should be noted that the explanations provided here are tentative. First, our Contact diversity measure specifically asked about the amount of interethnic/tribal contact, whereas previous MGM studies measured contact in general. Thus, our contact measure taps the frequency of activating one’s own ethnic/cultural identity, whereas general contact measures do not. This difference may have enabled Contact diversity to be related to EF above and beyond MGM for low or high SES individuals. Second, the relationships between Contact diversity and EF may be related to the felt positivity of intergroup contacts. Although both high and low SES individuals may be equally prejudiced against non-ingroup members^39^, high SES individuals have more financial resources to choose more interethnic contacts^40^, or reduce them if undesired, while low SES individuals have less resources to increase desired or decrease undesired contacts. This implies that the rate of negative contacts is kept low for high SES people when the number of contacts increases, but it would be difficult for low SES people. Chronic negative contacts would distress individuals, which lowers EF^41–43^. Some may argue that having too few desired contacts would also undermine EF by creating loneliness-related stress^44^, which should have nullified the relationship between contacts and EF for low SES people. Although we cannot fully rule out this possibility, there are two reasons why this explanation is unlikely. First, as mentioned above, our contact measure specifically asked about interethnic contacts rather than the overall contacts people had, which makes the measure less relevant to loneliness. This is caused by the fact that the number of overall contacts can vary independently from the number of interethnic contacts. For instance, an individual can have many contacts within their ethnic group, and thus feel less lonely, without necessarily having any contact with another ethnic group. Second, our regression model included MGM, which should control for the variances in felt loneliness and perceived social support derived from variations in MGM. Again, as the findings of Contact diversity were not predicted a priori, more studies are needed to directly replicate them and to better understand the processes.

More fundamentally, is there any potential explanation to what causes the link between MGM and EF? The present study is cross-sectional and thus we cannot establish the causality of the effects. Instead, the main objective of this study was to clarify an associative link between MGM and EF. However, our results can also point towards two potential explanations that will need future longitudinal or experimental studies to be tested. First, it could be speculated that the number of groups to which one belongs are the results of individual differences in EF. Individual differences in EF are thought to be determined by a combination of genetic, experiential, and contextual factors^45,46^. These individual differences would then become a constraint to all the daily tasks that would require them, including the task of flexibly updating WM contents as individuals alternate their identities based on their multiple social groups. If this explanation is true, then variations in individual EF capacities would have a direct impact on the number of social groups that an individual can afford to cope with.

A second explanation is derived from a framework of neuroplasticity. According to this framework, frequent cognitive stimulation can potentially boost neuroplasticity and lead to a facilitation of several cognitive systems, including EF^47,48^. This theoretical framework is supported by data suggesting that lifetime cognitive stimulation protects against cognitive ageing^49^ and data suggesting that cognitive training programs can facilitate neuroplasticity^50^. However, evidence against this framework also exists, in particular research suggesting that cognitive training has limited, if any, benefits on general cognitive function^51^.

Additional evidence for the neuroplasticity framework comes from longitudinal studies indicating that enhanced social activity can lead to improved cognitive outcomes. For example, Glei et al. (2005)^52^ found social activities defined as playing games (e.g., chess), socializing with friends, and/or participating in groups helped preserve cognitive function of elderly Taiwanese while weekly social contacts did not. Barnes et al. (2004)^53^ demonstrated that both social contacts and social engagement (social and productive activities) reduced a rate of cognitive decline among the elderly. C. Haslam, Cruwys, and Haslam (2014)^23^ showed that group engagement (participating in cultural and community activities and the number of group memberships) uniquely predicted subsequent cognitive function in a sample of British participants above 50 years old while individual engagement (frequency, quality, and the number of contacts) did not.

If we apply an explanation based on neuroplasticity to our results, it could be posited that as individuals become members of social groups and identify with them, they will have to frequently update representations and coordinate behaviours specific to each of these social groups. Therefore, a high level of MGM could in itself become a source of cognitive stimulation, which could in turn potentially facilitate neuroplasticity in EF systems. These two explanations are not mutually exclusive, as both processes could potentially coexist and jointly contribute to the MGM-EF relationship. However, we acknowledge that our study cannot disentangle these two explanative frameworks. Only future research using longitudinal or experimental designs would be able to fully test these two explanations.

A distinction that may be important for future studies is the one between *multiplicity* and *distance* between groups. This study focused on the multiplicity of groups people belong to but not on the distance between these groups. In the introduction, we gave the example of an Asian descendant living in the US who should have very “distant” identities (different norms and values) as an Asian and as a Westerner. Having multiple group memberships, however, does not always lead to having distant identities. Research examining the relationship between dual identities and creativity argues that people belonging to more distant groups show higher creativity^54^ while studies on MGM showed that mental health is better when a new group membership is compatible (similar norms and values) with one’s social background^29^. Would distance in norms and values moderate the association between MGM and EF? This is an interesting and important question for future research. These considerations raise the possibility that the association between MGM and EF might be moderated by the distance in norms and values, and future research will be needed to examine this hypothesis.

Finally, it is important to point out that our results involving SES led to a number of interesting possibilities. First, our study shows that SES is correlated with both Contact diversity and MGM. In other words, in our sample, people with a high SES tend to be in contact with more diverse people, and they also belong to more social groups and thus derive more multiple identities from this situation. These results are not surprising and are consistent with the suspicion that SES is linked to the availability of more resources that lead to a more varied and diverse social life on average^29^. A high SES is typically linked to more resources to belong to different groups (hobbies groups, mobility, travelling, etc.), which can lead to a greater variety of interactions with other people. While these relationships need to be tested with more samples from diverse cultures, this finding is important given what is known about the benefits of social contact and group membership^3,6,23,52,53^, as well as the possible negative outcomes diverse contacts could bring about, as the present study suggested.

## Conclusion

This cross-sectional study is the first to show that belonging to multiple social groups is related to individual differences in a canonical EF task. These findings can contribute to a better understanding of the cognitive characteristics of individuals with social lives that lead them to juggle between multiple social identities, and it sets the path for future longitudinal studies needed to establish the causal links underlying these findings. In addition, our findings are consistent with prior work on the relationship between MGM and creativity^19^, and past and current findings suggest that EF may be a key mechanism through which MGM relates to cognitive performance. Furthermore, this study also unveiled relationships between socioeconomic status and both the diversity of social contact and the level of MGM of an individual. These findings are important for policy makers because they suggest that socioeconomic inequalities could extend to the benefits and drawbacks of living a diverse and complex social life.

## Method

### Participants

The initial sample of 521 adults (311 females) who could at least read numbers was invited to our study using convenience sampling method targeting both low and middle-to-upper SES communities in Surabaya. Their ages were between 18 and 73 (mean age = 35.61, *SD* = 13.15). The data and main analysis script can be retrieved from this anonymous link: https://osf.io/z6t5g/?view_only=b571d0f839c44caabb95d21d1557e49c. Forty-seven participants did not fully complete the N-back tasks, and 71 participants did not have enough trials (< 36) of the 3-back task, mostly because they did not understand the instruction (e.g., not responding to non-target trials), and one additional participant did not reach the 30% accuracy threshold for the 3-back task (See N-back Performance in the Results section). One additional participant’s value of family income was an outlier (extremely low). Six additional participants did not report at least one of the five key explanatory variables included in our data analysis. Thus, our final sample for regression analyses included 395 participants (228 females, 18 to 73 years old, mean age = 35.14, *SD* = 13.06). Statistical power analyses established that this sample size had sufficient power (0.98) to detect a small to medium effect size correlation, r = 0.2^55^ with α = 0.05 (G*Power 3.1.9.4^56^). They were citizens of Surabaya, the second largest city in Indonesia located in the Eastern part of Java Island. They were sampled from different districts of the city (Dharmawangsa, Gading, Jagir, Kedinding, Kedung Cowek, Keputih, Menur Pumpungan, Pacar Keling, Ploso, Pegirian, Rangkah, Sidodadi, Sidotopo, Simokerto, Simolawang, Wonokromo, and Wonokusumo), in order to achieve a reasonable spread of SES. Low-income communities were approached thanks to social programs delivered by a public university in the area. In our final sample, 89.1% of participants were from Javanese background, 7.5% were from Madurese background, and the rest were from various other ethnic backgrounds (e.g., Chinese, Balinese, Sundanese, etc.). 89.7% of the sample’s participants were Muslims, 4.68% were Protestants, 4.3% were Christians, 1.3% were Hindus, and 0.3% were Buddhists. They received 25,000 Indonesian Rupiah (~ 1.65 USD in 2018) in exchange of their time. The median monthly household income was 5,500,000 IDR (~ 363 USD; range = 260,000–250,000,000 IDR, ~ 17.16–16,500 USD) and a mean for per capita household monthly income (PCHI) was 3,166,232 IDR (~ 208.97 USD) (*SD* = 5,100,042). The highest PCHI was 50,000,000 IDR, top 20% was 4,295,238 IDR, top 40% was 2,000,000 IDR, top 60% was 875,000, top 80% (bottom 20%) was 400,000 and up IDR, and the lowest was 65,000 IDR. PCHI was skewed, and thus, we transformed this variable with natural log.

### Measures

The present study was conducted as a part of a broader project on the psychological correlates of poverty in South-East Asia. We report here only the variables that are relevant to the research questions tackled by the present study: SES, multiple group memberships (MGM), Contact diversity, and EF as well as age and gender.

#### SES

SES was estimated with two measures: Per Capita Household Income (PCHI) and education level. PCHI is a classical measure of SES^57,58^. It was defined as monthly household income divided by the number of household members. Therefore, participants were asked to disclose their monthly household income, defined as the sum of each participant’s personal income and the income of anyone who lives and shares expenses with them. They were also asked to disclose the number of household members, defined as the number of people who contribute to and/or depend on their household income. Participants’ education levels were recorded by asking whether they attended a college or not. We chose this measure as most of the participants in the past MGM studies attended a college or more (for example^20^). In the current final sample, 215 participants attended a college or above and 180 did not attend a college, indicating that the current sample came from both low and high SES backgrounds. We standardized these measures first and then averaged to indicate participants’ SES.

##### Multiple group membership (MGM)

We used a measure developed by C. Haslam et al. (2008)^59^ and used by others examining effects of MGM^19^. This measure included four items: “I belong to many different groups”, “I join in the activities of lots of different groups”, “I have friends who are members of many different groups”, and “I have strong ties with lots of different groups” (Cronbach’s *α* = 0.920). Before answering the questions, participants were told that groups could be a work group (a group of people you work with), a professional group (for example, "teacher", "engineer"), a social group (defined broadly, e.g., a social gathering group), a sports group (such as a football club), or any specific category based on age, gender, or occupation. Participants answered on a 7-point Likert scale: 1 = strongly disagree and 7 = strongly agree and averaged across the items.

##### Contact diversity

We adapted three items from the past research^60,61^ that estimate the frequency of interactions between the respondents and other people from different ethnic groups^60,61^. Specifically, the English translation of our items is the following: “I spend a lot of time to do things with friends of different ethnic or tribal groups,” “Friends of different ethnic or tribal friends often come to my home,” and “I often visit homes of different ethnic or tribal friends” (Cronbach’s *α* = 0.866). Participants answered on 7-point scale: 1 = strongly disagree and 7 = strongly agree. These three items were averaged to create a composite score. The *Bahasa Indonesia* translation “ethnic” and “tribe” that we used, *etnis/suku*, refers to social groups organized by ethnic divisions, to be compatible with previous research on intergroup contact^60^.

### N-back task

**Figure 4:**
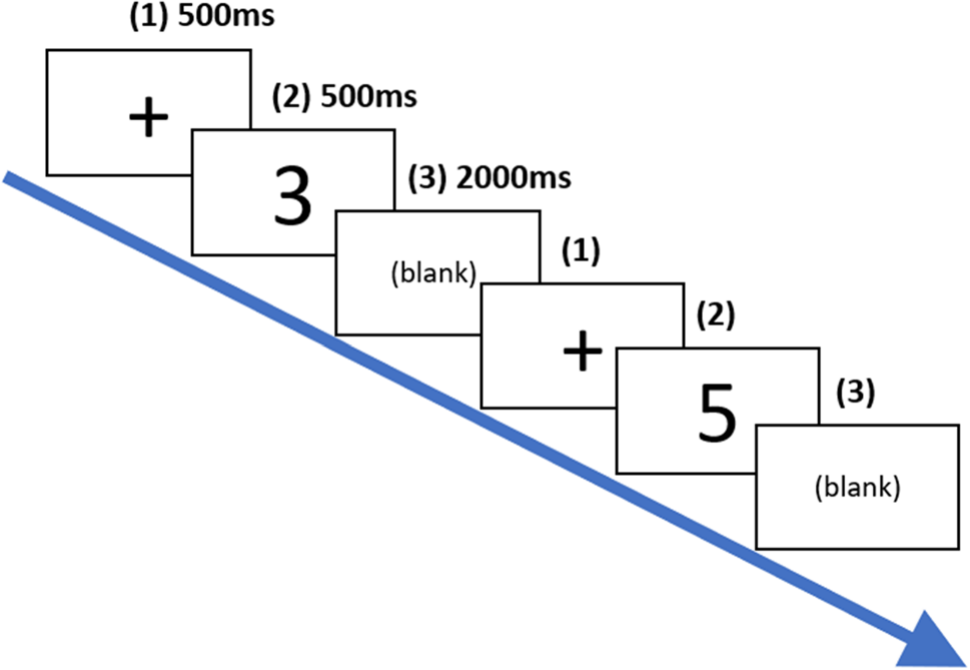
The procedure of the N-back tasks.

In order to assess EF, we used a 3-back working memory task. This task is considered as a canonical EF task, and it is often used to probe individual differences in EF^32,62,63^ and often thought to tap the *updating* subprocess of executive control^64^. In this task, participants were shown on computer screen sequences of digits displayed one at a time. For each digit, participants had to decide whether or not it matched the one displayed three digits ago by pressing a button. There were three blocks and each block had 27 trials. Within each trial, (1) a fixation point (black cross on white background) was displayed for 500 ms; (2) a black font digit that was drawn from between 1 and 9 was displayed for 500 ms over a white background; (3) a blank screen was presented for 2 s (Fig. 4). Participants were allowed to respond via a key press during (3), and the blank screen remained even after participants made a response. All testing was done on laptops using Psychopy2^65^, and participants only needed to use two keys (corresponding to target and nontarget responses) clearly indicated with a coloured sticker. Since the first three trials were always non-target trials (these three digits did not have “the one displayed three digits ago”), we counted the numbers of hits and correct rejections out of only 24 trials in each block and computed *d’*^32,66^. Consistent with previous research, one third of the 24 trials were target trials^62^. Compared to the samples usually tested in cognitive psychology studies, our sample was unfamiliar with psychological testing. Therefore, they were extensively briefed on how the N-back task works, and they went through a long practice run of 22 familiarization trials with the option to repeat in order to make sure that they understood the requirements of the task. Before the 3-back task, participants also took part in 1-back task, a simpler variant of the N-back task in which participants had to decide each current digit matched the last digit that was displayed. In this task, there were three blocks and each block had 25 trials. The rest of the procedure and the duration of stimuli were identical to the 3-back task.

### Procedure

The data collection procedure consisted of two phases: a structured interview and EF tasks. The structured interview involved face-to-face interactions with every individual participant. During the interview, data collectors read aloud predetermined questions and presented Likert scales visually to each individual participant. They were then prompted to respond by indicating the number corresponding to their choice in each scale. For every question, clarifications were provided when needed. We chose a structured interview format instead of a questionnaire format because many participants were not familiar with questionnaires, and because we anticipated varying levels of literacy in our sample. A half of the participants started with the structured interview while the other half started with the EF tasks.

Data was collected by 20 fully trained research assistants who were all Indonesians and either had completed or were in the last (4th) year of completing a degree in one of the main public universities in Indonesia. They worked in pairs or in groups and they were all fluent in the main national language, *Bahasa Indonesia*. Although this is a *lingua franca* widely used all throughout Indonesia, some of our participants were more comfortable with other local languages. To address this issue, most of our data collectors could speak an additional local language, *Javanese* or *Madurese*, in such a way that structured interviews were always conducted in participants’ preferred language. The data collection took place either in participants’ homes, at their workplaces, at community centers, or in designated rooms of the university one of the authors was affiliated. The protocol of this study was developed in accordance with the Declaration of Helsinki and was approved by Monash University’s Human Research Ethics Committee (Melbourne, Australia), and every participant signed a standard informed consent form as per the rules of the committee.

### Ethical approval

The protocol of this study was approved by Monash University’s Human Research Ethics Committee (Melbourne, Australia) and every participant signed a standard informed consent form as per the rules of the committee.

## Data Availability

The data and main analysis script as well as supplementary figures can be retrieved from this anonymous link: https://osf.io/z6t5g/?view_only=b571d0f839c44caabb95d21d1557e49c.
